# PrescrAIP: A Pan-European Study on Current Treatment Regimens of Auto-Immune Pancreatitis

**DOI:** 10.3389/fmed.2020.00408

**Published:** 2020-08-05

**Authors:** Marco Lanzillotta, Olof Vinge-Holmquist, Kasper A. Overbeek, Jakob L. Poulsen, A. Fatih Demirci, Peter Macinga, Matthias Löhr, Jonas Rosendahl

**Affiliations:** ^1^Unit of Immunology, Rheumatology, Allergy and Rare Diseases (UnIRAR), San Raffaele Scientific Institute, Milan, Italy; ^2^Department of Gastroenterological Surgery, Akershus University Hospital, Loerenskog, Norway; ^3^Department of Gastroenterology & Hepatology, Erasmus University Medical Center, Rotterdam, Netherlands; ^4^Centre for Pancreatic Diseases, Department of Gastroenterology & Hepatology, Aalborg University Hospital, Aalborg, Denmark; ^5^Department of Internal Medicine, Marmara University Research and Education Hospital, Istanbul, Turkey; ^6^Department of Gastroenterology and Hepatology, Institute for Clinical and Experimental Medicine, Prague, Czechia; ^7^Gastroenterology and Hepatology, Gastrocentrum, Karolinska University Hospital, Karolinska Universitetssjukhuset, Stockholm, Sweden; ^8^Department of Internal Medicine I, Martin Luther University, Halle (Saale), Germany

**Keywords:** autoimmune pancreatitis, IgG4 (autoimmune pancreatitis), glucococorticoids, cohort studies, IgG4

## Abstract

**Introduction:** Treatment of autoimmune pancreatitis (AIP) is based solely on consensus and has yet to become standardized. Consequently, therapeutic regimens vary greatly between countries and centers, and largely depend on the experience of the physician. At this moment, the optimal regimen for inducing disease remission and preventing relapse is unknown.

**Objectives:** The primary objective of this study is to describe current treatment regimens used in Europe, and to compare their effectiveness in inducing remission and preventing and treating relapse. The secondary objectives are: to identify risk factors for relapse; to assess the diagnostic accuracy of the Unified-AIP criteria; to assess the performance of the M-ANNHEIM score for predicting relapse; and to assess long-term outcomes including pancreatic exocrine insufficiency and pancreatic cancer.

**Methods:** This is an international, retrospective, observational cohort study, performed in over 40 centers from 16 European countries. Eligible are all patients diagnosed with AIP from 2005 onwards, regardless of the used diagnostic criteria. Data on study subjects will be retrieved from the hospital's electronic medical records and registered with a standardized, web-based, electronic case report form (eCRF). To compare the effectiveness of treatment regimens in inducing remission, preventing relapse, and treating relapse, subjects will be stratified in groups based on: type of therapy; initial therapy dose; cumulative therapy dose; therapy tapering speed and duration; and having received maintenance therapy or not.

**Ethics and Dissemination:** Ethical and/or institutional review board approvals are obtained by all participating centers according to local regulations. The study complies with the General Data Protection Regulation (GDPR). All manuscripts resulting from the study will be submitted to peer-reviewed journals.

**Conclusion:** This is the first pan-European retrospective registry for AIP. It will produce the first large-scale data on treatment of European patients with AIP, providing answers on the use and effectiveness of treatment regimens. In the future, this collaboration may provide a network for continuation into a prospective European registry.

## Introduction

Autoimmune pancreatitis (AIP) has recently been recognized as an immune-mediated disease of the pancreas with distinct features (1). It is a rare disease with an annual incidence of ~3.1/100,000, which varies substantially between geographical regions (2). To date, two types of autoimmune pancreatitis have been described (1). Type 1 AIP is the pancreatic manifestation of IgG4-related disease (IgG4-RD). It shares clinical and histological hallmarks with IgG4-RD, namely increased serum Immunoglobulin G4 (IgG4) levels, dense storiform fibrosis, and IgG4-positive plasma cell infiltration of the affected organ (3). Type 2 AIP is known as idiopathic duct-centric pancreatitis and is characterized by neutrophil-mediated duct destruction, in the form of granulocytic epithelial lesions (GEL) (4). Both types can cause abdominal pain and jaundice and can ultimately lead to chronic pancreatitis (1). This in turn, might even increase the risk of developing pancreatic cancer (5). Yet, the incidence of these complications has not been clearly established.

In the last two decades, several efforts have been made to establish diagnostic criteria (1, 6–8), which are all based on the combination of clinical, serological, and pathological features. Nevertheless, AIP cases are still missed or even mistaken for pancreatic ductal adenocarcinoma (9, 10). As such, sequelae of chronic pancreatitis with endocrine and exocrine insufficiency can develop or the diagnosis of type 1 and type 2 AIP is made after unnecessary surgery (11). In addition, a discrete percentage of AIP cases does not fulfill diagnostic criteria for type 1 or type 2 AIP and thus are referred to as Not-Otherwise-Specified (NOS) AIP (1).

All AIP subtypes respond dramatically to steroid treatment (up to 99% in the different cohorts) (11–14), but the optimal dose to induce remission remains controversial. Reported induction doses have ranges between 30 and 60 mg daily (11–14) Recently, other therapeutic options, that induce B-cell depletion, have also been employed in inducing AIP remission, with promising results (15, 16). In a cohort from France, the reported efficacy was 94% with two infusions given in most patients (17). However, in spite of the dramatic response to initial treatment, the risk of relapse within 1 year from disease remission ranges between 30 and 50% (12), being higher in type 1 than in type 2 AIP patients (12). Several risk factors for AIP relapse have been proposed, but have not been validated prospectively (11, 12, 18, 19). Due to the high frequency of relapse after induction, some authors recommend maintenance treatment with low-dose steroids or immunomodulators, but the patient group that would benefit from maintenance is currently unknown (20–26).

Thus far, data on the epidemiology and natural history of AIP are still scant due to its rarity and its relatively recent appraisal. Most of the data comes from Asian or North-American cohorts, while data on large European cohorts are lacking. As a consequence, treatment options applied in Europe are largely based on retrospective studies from Asian and North-American patients.

We established the PrescrAIP (A Pan-European Study on Current Treatment Regimens of Auto-immune Pancreatitis) study network to retrospectively describe the current status of AIP treatment in Europe on a large scale. In addition, our effort will create the opportunity for a subsequent international prospective registry that will be able to provide definite answers in the future. In particular, in this retrospective multicentre study, we aim to define and compare the AIP treatment regimens used throughout different European centers, highlighting their differential impact on disease remission and long-term outcomes. These results will foster our knowledge of this rare disease, yielding to better patient care.

## Objectives

The primary objective of this study is to describe AIP treatment regimens across Europe, and to compare the effectiveness of treatment regimens in inducing remission, preventing and treating relapse. The secondary objectives include identifying risk factors for relapse, assessing the diagnostic accuracy of the U-AIP criteria (6), assessing the performance of the M-ANNHEIM score for predicting relapse (19), and assessing long-term outcomes including pancreatic exocrine insufficiency and pancreatic cancer. Thirdly, we are aiming to assess whether standard treatment was altered due to pre-existing diabetes mellitus, determine the prevalence of diabetes mellitus before and after steroid treatment, assess glycemic control in patients with diabetes mellitus and to describe the clinical, radiological and pathological characteristics of a cohort of patients diagnosed with AIP through pancreatic resection.

## Methods

### Study Design

This is an international, retrospective, observational cohort study including all AIP patients. We used the Pancreas2000 framework (www.pancreas2000.org) to create a study network starting with the six centers in the PrescrAIP core group. Additional European centers with expertise in the treatment of AIP patients have been recruited, accumulating to a total of 44 collaborating centers.

### Study Population

Every patient with an AIP diagnosis (type 1, type 2 or NOS-AIP) will be included, regardless of diagnostic criteria used (U-AIP, HISORt, ICDC). Patients with AIP diagnosed prior to 2005 will be excluded due to lack of uniformity in diagnostic standards.

### Setting

**Figure 1:**
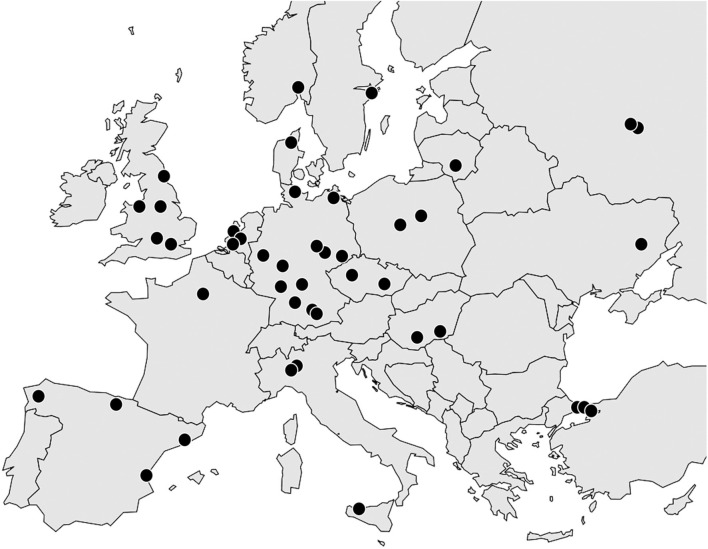
European centers (dots) involved in the PrescrAIP Study.

The collaboration involves a large number of European centers, most of which are academic hospitals (Figure 1). Starting with the eight centers of the PrescrAIP core group, the study now encompasses centers in Germany (12), the United Kingdom (5), the Netherlands (3), Turkey (3), Italy (3), Spain (3), Norway (2), Poland (2), Russia (2), Hungary (2), Czech Republic (2), Lithuania (1), Ukraine (1), Denmark (1), Sweden (1), and France (1).

### Data Collection

Patient data will be collected from the hospitals' medical records. Variables were selected to answer the research questions and meet the objectives of the study. Variables will be recorded in a REDCap-based (https://www.project-redcap.org) electronic datasheet (electronic case record form, eCRF), hosted by The North Denmark Region. The principal investigator of each site will ensure that the data in the eCRF are accurate, complete and legible. REDCap is a secure, online application designed to support data acquisition and storage by providing a shapeable interface for validated data entry.

### Assessment Variables

At the time of inclusion, all relevant variables will be recorded in the eCRF. These will include variables on demography and epidemiology, disease characteristics (radiological, laboratory and clinical), the set of diagnostic criteria employed, treatment (type, dose, duration), and short and long-term clinical outcomes. The complete variable list and definitions are reported in Appendix 1. AIP subtypes will be defined following the analysis of the above-mentioned variables. Given that patients with elevated serum IgG4 but without other organ involvement or biopsy sample can potentially be misdiagnosed, a subgroup with these characteristics will be created. Then a sensitivity analysis with and without excluding this subgroup will be performed to evaluate potential differences in the outcomes.

### Study Endpoints

Our primary endpoints consist of remission of disease (defined as the absence of clinical symptoms and the resolution of pancreatic abnormalities on imaging), relapse of disease, relapse rates compared between patients with a low or a high dose regimen, cumulative maintenance therapy dose and relapse-free survival time. Our secondary study endpoints are gold standard diagnostic tools of AIP compared to U-AIP, the prevalence of pancreatic exocrine insufficiency and the cumulative incidence of pancreatic ductal adenocarcinoma. The diagnosis of diabetes mellitus [as defined by the American Diabetes Association (27)] either before or after steroid treatment is our tertiary study endpoint.

### Statistical Analysis

Continuous, non-normally distributed values will be presented as median and interquartile range (IQR), unless otherwise specified. Discrete variables will be presented as frequency (percentage). Normal distribution of continuous variables will be assessed with the Kolmogorov–Smirnov algorithm. Normally distributed variables among the different groups will be compared using the Student's *t*-test. Non-normally distributed variables will be compared using the Mann–Whitney *U*-test. Categorical variables will be analyzed by the Fisher's exact test or chi-square test.

To compare remission rates, relapse rates, relapse-free survival and long-term outcomes between participants, we will stratify participants according to the date of diagnosis, meeting the different diagnostic criteria, the type of treatment, the glucocorticoids starting dose, the cumulative dose, tapering speed, and treatment with maintenance treatment.

Moreover, to assess the impact of the treatment effect being part of the diagnostic criteria, we will perform sensitivity analyses comparing the study outcomes between those classified as AIP regardless of the steroid trial, and those in whom the diagnosis was dependent on the steroid trial. Sensitivity analyses will also be performed to compare the study outcomes including or excluding individuals that do not meet any available diagnostic criteria, and those classified as NOS-AIP.

Kaplan-Meier curves will be used to assess time-to-relapse. Time-to-relapse will be compared between subgroups using the log-rank test. Univariable and multivariable analyses with a backward selection procedure will be performed to identify possible predictors for relapse, based on a Cox-proportional hazards regression model. A significance threshold of *P* < 0.05 will be used.

Data from national pancreatic cancer registries will be used to determine age- and sex-specific incidence rates for pancreatic ductal adenocarcinoma. Standardized incidence ratios (SIRs) will be then calculated by obtaining the ratio of the observed to the expected number of cases, and 95% confidence intervals (95% CIs).

## Ethics and Disseminations

This study was reviewed and approved by the ethics committees of the centers in the PrescrAIP core group, namely those at the San Raffaele Scientific Institute (Milan, Italy), the South East Regional Committee for Medical and Health Research Ethics (Oslo, Norway), the Erasmus University Medical Center (Rotterdam, The Netherlands), the Aalborg University Hospital (Aalborg, Denmark), the Marmara University School of Medicine (Istanbul, Turkey), the Institute for Clinical and Experimental Medicine (Prague, Czech Republic), the Karolinska University Hospital (Stockholm, Sweden), and the Martin Luther University Halle-Wittenberg (Halle, Germany). In addition, participating centers had the protocol reviewed and approved wherever required by local regulations.

Patients' data will be collected retrospectively from pre-existing electronic patient records. Study data will then be collected and managed using a REDCap database hosted at Aalborg University Hospital, North Denmark Region, Denmark. All data will be coded (pseudonymized). The key to the coded data will be stored locally in the participating site, in a password-protected file controlled by the Principal Investigator, and separately from any research data. For the German centers data will be anonymized immediately. When data is exported from the REDCap system for analysis, the data will be made completely unidentifiable and potential identifier variables will be removed. Additional processing of already collected data for the purpose of scientific research is exempt from specific consent according to Articles 5(1)(b) and 89(1) of the General Data Protection Regulation (GDPR). The study adheres to the Declaration of Helsinki. No patient will be exposed to any inconvenience in relation to the present study because all data are obtained retrospectively. The findings of the study will be published in a peer-reviewed journal and disseminated at national and international conferences.

## Discussion

AIP has only been acknowledged as a discrete entity in the last 20 years (1), even though the first reports date back to the 1960's (28). In the last decade, international efforts led to the creation of several sets of diagnostic criteria (1, 6–8), definitely raising awareness on AIP and providing guidelines for its treatment. Despite significant progress in the field, key questions related to the pathophysiology, diagnosis, treatment, and treatment-related complications of AIP remain unanswered. Therefore, AIP still poses a clinical challenge and the diagnosis is still overlooked. In particular, the role and efficacy of glucocorticoids in the induction of remission has been widely accepted and reported, but the optimal starting dose as well as tapering speed is far from being elucidated. In addition, several risk factors for relapse have emerged recently, but only few derive from large cohorts and none have been validated prospectively (11, 12, 18, 19). Finally, long term outcomes in terms of pancreatic exocrine and endocrine dysfunction, as well as incidence rates of malignancies, have rarely been addressed, ultimately impacting on patients' nutritional status and survival (29–31).

To a certain extent, the scarcity of data available can be linked to the low incidence of AIP. Together with its relatively recent appraisal, this complicates the implementation of adequately powered randomized controlled trials. Through an international European-based, multicenter effort, we plan to shed light on this multifaceted condition in order to develop evidence-based treatment strategies in the future. As such, our aim is to collect all available AIP cases, reporting clinical, laboratory, and treatment-related features in order to create an accurate picture of the current European AIP management and to obtain novel data on the natural history of AIP in Europe. Stemming from these, new questions may be formulated and evaluated in a prospective continuation of the present study. In addition, as mentioned above, due to the relatively young age of AIP long-term sequelae have often been overlooked by seminal studies in this field. Yet, a better knowledge in the development of pancreatic exocrine and endocrine (type 3c diabetes or “pancreatogenic” diabetes) insufficiency or treatment related diabetes is warranted in order to deliver better quality of life.

This project has several strengths. Based on a multicentre cohort, this pan-European AIP study will provide a large dataset, dealt by field experts. Indeed, the vast majority of AIP reports derive from North-American or Asian cohorts, with most likely distinct genetic or environmental features that might not be shared by European AIP patients. Therefore, our study will plausibly provide a more homogenous AIP population thus far not included in recent trials. Due to the rarity of AIP, multicentric collaboration offers the unique possibility of obtaining more reliable results since larger patients' cohort can be analyzed. Moreover, the electronic datasheet will guarantee data quality and safety and will also stimulate data homogeneity across the various very heterogeneous countries and practices.

The study does not come without limitations, it being a descriptive retrospective study. There is a risk of the clinical and diagnostic guidelines being applied differently throughout the recruitment period. To mitigate this, we have collected clinical symptoms, laboratory and radiological findings rather than using the derivative AIP subtyping of the clinician. For example, as in most institutions pancreatic biopsies are not performed, seronegative type 1 AIP confined to the pancreas could be misdiagnosed as type 2. Conversely, atypical type 2 AIP with elevation of serum IgG4 might be classified as type 1 AIP. By subgrouping patients according to clinical, histopathological (where available) and radiological findings we aim to better judge the AIP subtype of the patient we aim to better judge the AIP subtype of the patient and increase transparency and uniformity of the AIP diagnoses made. As the response to a steroid trial might in some cases establish the diagnosis of AIP incorrectly, we will perform a subgroup analysis in patients in whom the AIP diagnosis was based on the successful steroid trial. Hereby, we will be able to estimate the effect of this group on the overall results of our analysis. In addition, we will be enabled to perform sensitivity analyses in that distinct subgroups, as described above. Lastly, many patients have a long follow-up, which should strengthen the true AIP diagnosis.

In conclusion, our work will provide detailed description on the natural history and management of AIP in Europe, representing a unique framework for future prospective studies that may be able to provide definite answers to the questions that remain after the current retrospective evaluation.

## Author Contributions

All named authors contributed to planning, conduct, and reporting of the work.

## PrescrAIP Study Group

1. Aalborg University Hospital, Aalborg, Denmark: **Poulsen JL, Olesen SS, Drewes AM**
2. Acibadem University Hospital, Istanbul, Turkey: **Sisman G**
3. Akershus Universitetssykehus, Oslo, Norway: **Vinge-Holmquist O**
4. Amsterdam UMC, location AMC, Amsterdam, The Netherlands: **Beuers UHW, Sindhunata D**
5. A.S. Loginov Moscow Clinical Scientific Center, Moscow, Russia: **Kiriukova M, Bordin DS**
6. Beaujon Hospital, Clichy, Paris University, Paris, France: **Rebours V, Levy P**
7. Children's Memorial Health Institute, Warsaw, Poland: **Oracz G**
8. Christian-Albrechts Universität zu Kiel, Kiel, Germany: **Arlt A**
9. Donetsk National Medical University, Donetsk, Ukraine: **Gubergrits N**
10. Erasmus University Medical Center, Rotterdam, The Netherlands: **Overbeek KA, Bruno MJ, Cahen DL, Buijs J**
11. Hospital of Lithuanian University of Health, Kaunas, Lithuania: **Povilas I, Valantiene I**
12. Hospital Universitari Dr. Peset, Valencia, Spain: **López-Serrano A**
13. Hospital Universitario Cruces, Barakaldo, Spain: **Mart**í**nez-Moneo E**
14. Humanitas Research Hospital, Milan, Italy: **Auriemma F, Preatoni P, Carrara S**,
15. Institute for Clinical and Experimental Medicine, Prague, Czech Republic: **Macinga P, Hucl T**
16. Istituto Mediterraneo per i Trapianti e Terapie ad Alta Specializzazione (ISMETT), Palermo, Italy: **Barresi L**
17. Istanbul University, Faculty of Medicine, Istanbul, Turkey: **Akyuz F**
18. Karolinska University Hospital, Stockholm, Sweden: **Löhr M, Vujasinovic M, Nikolic S, Haase S**
19. Klinikum der Universität München, München, Germany: **Mayerle J, Goni E, Beyer G, Schönermarck U, Miksch R, Werner J**
20. Marmara University Hospital, Istanbul, Turkey: **Duman D, Demirci AF**
21. Medical Faculty Mannheim, Heidelberg University, Mannheim, Germany: **Schneider A, Hirth M**
22. Medical University of Łódz, Łódz, Poland: **Malecka-Panas E**
23. Newcastle Upon Tyne Hospitals, Newcastle, UK: **Nayar M, Vila J**
24. Oxford University Hospitals NHS Trust, Oxford, UK: **Culver EL**
25. Rikshospitalet, Oslo, Norway: **Vinge-Holmquist O**
26. San Raffaele Scientific Institute, Milan, Italy: **Lanzillotta M, Dagna L, Capurso G, Falconi M, Della Torre E**
27. Sechenov University, Moscow, Russia: **Okhlobystin AV, Kardasheva SS, Gonik MI**
28. Sheffield Teaching Hospitals, Sheffield, UK: **Hopper A, Jalal M**
29. Technische Universität München, München, Germany: **Algül H, Rasch S**
30. Universitätsklinikum C.G. Carus, Dresden, Germany: **Hampe J, Hinrichs N**
31. Universitätsklinikum Erlangen, Erlangen, Germany: **Vitali F**
32. Universitätsklinikum Essen, Essen, Germany: **Kahraman A**
33. Universitätsklinikum Greifswald, Greifswald, Germany: **Lerch MM, Aghdassi A, Frost F**
34. Universitätsklinikum Halle, Halle, Germany: **Rosendahl J**
35. Universitätsklinikum Leipzig, Leipzig, Germany: **Beer S, Hollenbach M**
36. Universitätsklinikum Marburg, Marburg, Germany: **Gress T, Grote T, Gallmeier E, Heuser DJ**
37. Universitätsklinikum Ulm, Ulm, Germany: **Kleger A, Backhus J**
38. University Hospital Brno, Brno, Czech Republic: **Kunovsky L, Trna J, Kala Z, Jabandziev P**
39. University of Szeged, Szeged, Hungary: **Czakó L, Darvasi E**
40. University Medical Center Utrecht, Utrecht, The Netherlands: **Vleggaar FP**
41. University of Liverpool, Liverpool, UK: **Greenhalf B, Halloran C**
42. Institute for Translational Medicine, Medical School, University of Pécs, Pécs, Hungary: **Hegyi P, Szentesi A**
43. University of Santiago de Compostela, Santiago de Compostela, Spain: **Dom**í**nguez-Muñoz JE, de la Iglesia D**

## Conflict of Interest

The authors declare that the research was conducted in the absence of any commercial or financial relationships that could be construed as a potential conflict of interest.
