# Supplementary material for: PrescrAIP: A Pan-European Study on Current Treatment Regimens of Auto-Immune Pancreatitis
Source: Front Med (Lausanne). 2020 Aug 5;7:408. doi: 10.3389/fmed.2020.00408 (PMC7419461; doi:10.3389/fmed.2020.00408)
Supplement: Supplementary file 1 [file Data_Sheet_1.PDF]

PrescrAIP - Appendix 1

VARIABLE LIST

PATIENT # *ID*

EPIDEMIOLOGY

AGE (at diagnosis)

value years

Race

☐ Caucasian

☐ Black

☐ Asian

☐ Latin-American

☐ Other

Gender

☐ Female

☐ Male

Occupation

☐ Blue-collar

☐ White-collar

☐ Not available

Tobacco smoking

☐ No

☐ Yes *How many?* cigarettes per day for *How many?* years

☐ Not available

Alcohol drinking

☐ No

☐ Yes *How many?* alcohol units er day for *How many?* years

☐ Not available

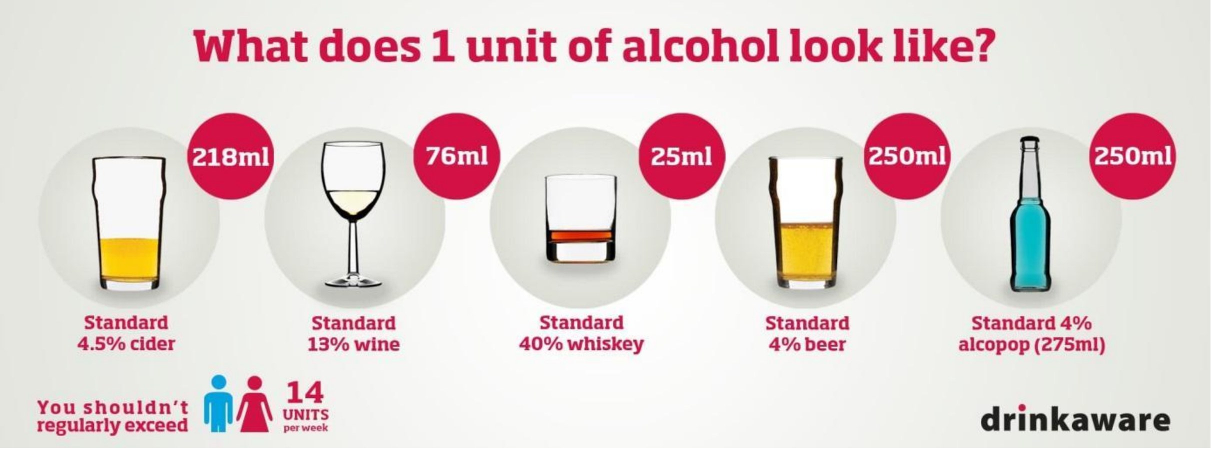

Allergy

☐ No

☐ Yes ☐ *Food* ☐ *Mold* ☐ *Dust* ☐ *Drug* ☐ *Insect sting*

☐ *Pet* ☐ *Skin* ☐ *Else* *Please specify*

☐ Not available

Comorbidities

History of pancreatitis before the diagnosis of AIP?

☐ No

☐ Yes ☐ *Acute* ☐ *Chronic*

How many? years before the AIP diagnosis

☐ Not available

IBD?

☐ No

☐ Yes ☐ *Crohn's disease* ☐ *Ulcerative colitis*

☐ *Indetermined colitis*

☐ Not available

Other autoimmune diseases?

☐ No

☐ Yes ☐ *Sjogren's syndrome* ☐ *Rheumatoid arthritis* ☐ *Sarcoidosis*

☐ *Autoimmune thyroiditis (NOT IgG4 related!)*

☐ *Else* *Please specify*

☐ Not available

DIAGNOSIS

Clinical manifestation

☐ Jaundice ☐ Abdominal pain ☐ Diarrhea ☐ Malaise ☐ Anorexia

☐ Nausea ☐ Night sweats ☐ Weight loss If yes, *How many?* kilograms

☐ Acute pancreatitis

M-ANNHEIM-AIP-ACTIVITY SCORE

☐ Ascites ☐ Vascular complications ☐ Cholangitis (bacterial, not IgG4 related)

PAIN REPORT ☐ no pain ☐ recurrent pain ☐ no pain with pain medication

☐ intermittent pain ☐ continuous pain

PAIN CONTROL ☐ none ☐ WHO step 1/2 ☐ WHO step 3

Date of the first symptom/sign onset

Year, month.

Radiological evidence for AIP

PARENCHYMAL IMAGING:

Modality:

☐ MR ☐ CT

Findings:

☐ Diffuse enlargement with delayed enhancement

☐ Segmental/focal enlargement with delayed enhancement

☐ Rim-like enhancement

☐ Else *Please specify*

☐ Not available

DUCTAL IMAGING

Modality:

☐ MRCP ☐ ERCP

Narrowing of main pancreatic duct without upstream dilatation:

☐ diffuse

☐ long *(>1/3 length of the main PD) OR multiple strictures without marked upstream dilatation*

☐ focal *(segmental/focal narrowing without marked upstream dilatation; duct size <5 mm)*

☐ Else *Please specify*

☐ Not available

M-ANNHEIM-AIP-ACTIVITY SCORE (IMAGING FINDINGS)

☐ Normal (= *main pancreatic duct <2mm, normal gland size and shape, homogenous parenchyma)*

☐ Equivocal (=*one of the following criteria: main PD enlarged {between 2 and 4mm}, slight gland enlargement {up to 2x normal}, heterogeneous parenchyma, small cavities {<10mm}, irregular ducts {including duct narrowing}, increased echogenicity of the main PD wall, irregular head/body contour)*

☐ Mild (=*two or more of the above listed criteria but normal main PD)*

☐ Moderate (=*two or more of the above listed criteria with main pancreatic duct abnormality {either enlargement between 2 and 4mm or increased echogenicity of the duct wall)*

☐ Severe (=*one or more of the following: large cavities {>10mm}, gross gland enlargement {>2x normal}, intraductal filling defects or calculi, duct obstruction, structure or gross irregularity, contiguous organ invasion)*

☐ Focal mass ☐ Focal enlargement ☐ Sausage

Histopathological evidence for AIP

☐ Periductal lymphoplasmacytic infiltrate without granulocytic infiltration

☐ Obliterative phlebitis

☐ Storiform fibrosis

☐ Granulocytic infiltration of duct wall (GEL)

☐ Granulocytic and lymphoplasmacytic acinar infiltrate

☐ Cytology only ☐ *suspected malignancy* ☐ *inconclusive*

☐ *negative for malignancy* ☐ *susp. AIP*

☐ Not reported

IGG4-POSITIVE CELLS

☐ Present *value* /HPF

☐ IgG4/IgG ratio *value* ☐ *Not available*

☐ Absent

☐ Else *Please specify*

☐ Not available

Serology

Serum IgG4 levels

Date of serology: *value* mg/dl *value* normal range of the lab

☐ Not available

Other Markers

☐ ANA ☐ RF ☐ anti-PBP ☐ ANCA ☐ AMA

☐ anti-TG ☐ anti-CA

☐ eosinophils count *Value* x 10^9/l

☐ total IgE *Value* kIU/l

☐ Else *Please specify*

Other organ involvement at the time of diagnosis:

☐ No

☐ Yes ☐ *sclerosing cholangitis* ☐ *sialadenitis* ☐ *thyroiditis*

☐ *orbital disease* ☐ *retroperitoneal fibrosis*

☐ *kidney disease* ☐ *(peri)aortitis*

☐ *lung disease (pneumonitis, pseudotumors)*

☐ *Else* *Please specify*

☐ SYMPTOMATIC ☐ ASYMPTOMATIC

☐ Not available

Applied diagnostic criteria

☐ ICDC ☐ HISORt ☐ U-AIP ☐ None/Clinical

Macroscopic type

☐ Focal *Head* ☐ *Body* ☐ *Tail* ☐

☐ Diffuse

Date of the diagnosis

Year, month.

Pancreatic surgery before the diagnosis of AIP?

☐ No

☐ Yes for indication: ☐ *cancer suspicion*

☐ *else* Please specify

TREATMENT

Pre-treatment characteristics

Weight Value kg

Height Value cm

DIABETES MELLITUS

☐ Absent

☐ Present DATE OF DIAGNOSIS year, month

TYPE ☐ DM 1 ☐ DM 2 ☐ Not available

DURATION ☐ new-onset DM (<6 months prior to diagnosis of AIP)

☐ known for >6 months

☐ Not available

TREATMENT ☐ diet

☐ oral antiglycemic medication

☐ insulin

HbA1c Value mmol/mol

☐ Not available

☐ Not available

PANCREATIC EXOCRINE INSUFFICIENCY

☐ Absent

☐ Present *DIAGNOSIS* ☐ fecal elastase Value ug/g

☐ steatorrhea

☐ breath test Value %

SEVERITY ☐ mild (no enzyme replacement)

☐ severe (enzyme replacement)

☐ Not available

Induction treatment

PREDNISONE

☐ No *REASON* ☐ Spontaneous relief of symptoms

☐ Surgery

☐ Contraindication for corticosteroids

☐ Randomized to other medication within RCT

☐ Diabetes

☐ Unknown

☐ Yes *DATE OF STARTING INDUCTION THERAPY* year, month

RESPONSE ☐ yes ☐ no

STARTING DOSE: *Value* mg/d for *Value* weeks

TAPERING

DOSAGE 2: *Value* mg/d for *Value* weeks

DOSAGE 3: *Value* mg/d for *Value* weeks

…

LOWEST DOSE: *Value* mg/d for *Value* weeks

DATE OF STOPPING INDUCTION THERAPY year, month

INDUCTION OF REMISSION? ☐ yes ☐ no

RITUXIMAB

☐ No

☐ Yes *REASON* ☐ RCT

☐ Contraindication for corticosteroids

☐ Unknown

DATE OF STARTING INDUCTION THERAPY year, month

DOSAGE *Value* mg/month in *Value* doses

RESPONSE ☐ yes ☐ no

INDUCTION REMISSION ☐ yes ☐ no

DATE OF STOPPING INDUCTION THERAPY year, month

Post-(induction) treatment characteristics

Weight *Value* kg

☐ Not available

Diabetes mellitus

☐ Absent

☐ Present *TREATMENT* ☐ diet

☐oral antiglycemic medication

☐ insulin

HbA1c *Value* mmol/mol

☐ Not available

☐ Not available

Pancreatic exocrine insufficiency

☐ Absent

☐ Present *DIAGNOSIS* ☐ steatorrhea

☐ breath test *Value* %

☐ fecal elastase *Value* ug/g

☐ Not available

Serum IgG4 levels

value mg/dl

Maintenance therapy

☐ No

☐ Yes *REASON* ☐ center experience

☐ high-risk features ☐ *sclerosing cholangitis*

☐ *serum IgG4 elevation* *value* mg/dl

☐ OOI treatment

☐ else *Please specify*

☐ unknown

Prednisone

☐ No

☐ Yes *DATE OF STARTING MT THERAPY* year, month

DOSAGE *Value* mg/day

DATE OF STOPPING MT THERAPY year, month

REASON FOR ENDING MT THERAPY ☐ Preplanned

☐ Relapse

☐ Side-effects

☐ Lack of compliance

☐ else *Please specify*

☐ Unknown

Azathioprine

☐ No

☐ Yes *DATE OF STARTING MT THERAPY* year, month

DOSAGE *Value* mg/day

DATE OF STOPPING MT THERAPY year, month

REASON FOR ENDING MT THERAPY ☐ Preplanned

☐ Relapse

☐ Side-effects

☐ Lack of compliance

☐ else *Please specify*

☐ Unknown

6-Mercaptopurine

☐ No

☐ Yes *DATE OF STARTING MT THERAPY* year, month

DOSAGE *Value* mg/day

DATE OF STOPPING MT THERAPY year, month

REASON FOR ENDING MT THERAPY ☐ Preplanned

☐ Relapse

☐ Side-effects

☐ Lack of compliance

☐ else *Please specify*

☐ Unknown

Methotrexate

☐ No

☐ Yes *DATE OF STARTING MT THERAPY* year, month

DOSAGE Value mg/week

DATE OF STOPPING MT THERAPY year, month

REASON FOR ENDING MT THERAPY ☐ Preplanned

☐ Relapse

☐ Side-effects

☐ Lack of compliance

☐ else Please specify

☐ Unknown

Mycophenolate Mofetil

☐ No

☐ Yes *DATE OF STARTING MT THERAPY* year, month

DOSAGE Value mg/day

DATE OF STOPPING MT THERAPY year, month

REASON FOR ENDING MT THERAPY ☐ Preplanned

☐ Relapse

☐ Side-effects

☐ Lack of compliance

☐ else Please specify

☐ Unknown

Rituximab

☐ No

☐ Yes *DATE OF STARTING MT THERAPY* year, month

DOSAGE Value mg/month in Value doses

DATE OF STOPPING MT THERAPY year, month

REASON FOR ENDING MT THERAPY ☐ Preplanned

☐ Relapse

☐ Side-effects

☐ Lack of compliance

☐ Else Please specify

☐ Unknown

Relapse

☐ No

☐ Yes *DATE OF RELAPSE* year, month

SERUM IGG4 LEVEL *value* mg/dl

ORGAN AFFECTED ☐ Pancreas

☐ Other *Please specify*

☐ Not available

Treatment of Relapse

Prednisone

☐ No

☐ Yes *DATE OF STARTING THERAPY* year, month

RESPONSE ☐ yes ☐ no

STARTING DOSE: *Value* mg/d for *Value* weeks

TAPERING

DOSAGE 2: *Value* mg/d for *Value* weeks

DOSAGE 3: *Value* mg/d for *Value* weeks

…

LOWEST DOSE: *Value* mg/d for *Value* weeks

DATE OF STOPPING THERAPY year, month

INDUCTION OF REMISSION? ☐ yes ☐ no

Rituximab

☐ No

☐ Yes *REASON* ☐ RCT

☐ Contraindication for corticosteroids

☐ Ineffectiveness of corticosteroids

☐ Unknown

DATE OF STARTING THERAPY year, month

DOSAGE *Value* mg/month in *Value* doses

RESPONSE ☐ yes ☐ no

INDUCTION REMISSION ☐ yes ☐ no

DATE OF STOPPING THERAPY year, month

Maintenance therapy after relapse

☐ No

☐ Yes

PREDNISONE

☐ No

☐ Yes *DATE OF STARTING MT THERAPY* year, month

DOSAGE *Value* mg/day

DATE OF STOPPING MT THERAPY year, month

REASON FOR ENDING MT THERAPY ☐ Preplanned

☐ Relapse

☐ Side-effects

☐ Lack of compliance

☐ else *Please specify*

☐ Unknown

AZATHIOPRINE

☐ No

☐ Yes *DATE OF STARTING MT THERAPY* year, month

DOSAGE *Value* mg/day

DATE OF STOPPING MT THERAPY year, month

REASON FOR ENDING MT THERAPY ☐ Preplanned

☐ Relapse

☐ Side-effects

☐ Lack of compliance

☐ else *Please specify*

☐ Unknown

6-MERCAPTOPURINE

☐ No

☐ Yes *DATE OF STARTING MT THERAPY* year, month

DOSAGE Value mg/day

DATE OF STOPPING MT THERAPY year, month

REASON FOR ENDING MT THERAPY
☐ Preplanned
☐ Relapse
☐ Side-effects
☐ Lack of compliance
☐ else Please specify
☐ Unknown

Mycophenolate Mofetil

☐ No

☐ Yes *DATE OF STARTING MT THERAPY* year, month

DOSAGE Value mg/day

DATE OF STOPPING MT THERAPY year, month

REASON FOR ENDING MT THERAPY
☐ Preplanned
☐ Relapse
☐ Side-effects
☐ Lack of compliance
☐ else Please specify
☐ Unknown

Rituximab

☐ No

☐ Yes *DATE OF STARTING MT THERAPY* year, month

DOSAGE Value mg/month in Value doses

DATE OF STOPPING MT THERAPY year, month

REASON FOR ENDING MT THERAPY
☐ Preplanned
☐ Relapse
☐ Side-effects
☐ Lack of compliance
☐ Else Please specify
☐ Unknown

MALIGNANCY

Pancreatic cancer

DATE OF DIAGNOSIS

year, month

HISTOLOGICAL EVIDENCE ☐ Yes

☐ No

☐ Not reported

LOCATION OF THE TUMOR

☐ Head

☐ Body

☐ Tail

SIZE OF THE TUMOR (MAXIMUM TUMOR DIAMETER)

Value cm

☐ Not available

SURVIVAL

☐ Alive ☐ Deceased

How many? months after diagnosis of cancer

☐ Not available

Other cancer

DATE OF DIAGNOSIS

year, month

TUMOR TYPE

Specify

SURVIVAL

Value months

☐ Not available

FOLLOW-UP

Date of latest contact

month / year
